# Eye dominance and minor physical anomalies in schizophrenia: relations between two biological markers of abnormal neurodevelopment

**DOI:** 10.3389/fpsyt.2023.1145578

**Published:** 2023-06-09

**Authors:** Katerina Akabalieva

**Affiliations:** Department of Psychiatry and Medical Psychology, Faculty of Medicine, Medical University Sofia, Sofia, Bulgaria

**Keywords:** schizophrenia, dysontogenesis, laterality, eye dominance, minor physical anomalies

## Abstract

**Background:**

To investigate the frequency of left eye dominance and minor physical anomalies (MPAs) in schizophrenia patients and control subjects and determine the interrelations of these two biological markers of neuronal dysontogenesis in schizophrenia.

**Subjects and methods:**

Three tests for eye dominance were administered as performance tasks, not preference questionnaires. Seven MPAs were examined. The sample consisted of 180 (98 schizophrenia patients and 82 control subjects). Several statistical methods for examining the eye tests separately and together were used to assess the difference in left-eyedness between schizophrenia patients and control subjects.

**Results:**

Left eye dominance is significantly higher in schizophrenia subjects. Left-eyed subjects are more stigmatized with MPAs. There is a strong positive correlation between left-eyedness and stigmatization with MPAs in schizophrenia patients.

**Conclusion:**

As hand dominance is under cultural pressure, eye dominance is culturally independent and is useful and reliable indicator of altered hemispheric lateralization. The significant positive correlations between left-eyedness and MPAs and the high concurrence of these biological markers in schizophrenia patients are a potent indicator of underlying aberrant neurodevelopment.

## Introduction

The **lateralization of brain function** represents the tendency of specialization to one side of the brain or the other of some neural functions or cognitive processes (1). Although the two hemispheres appear with similar gross anatomy, a different model of neural connectivity lies behind the different specialized function in each hemisphere. Hand, foot, eye, ear dominance and linguistic lateralization are an expression of functional brain asymmetry (2). Some authors pointed that cerebral asymmetry is a part of the evolution of humans from earlier primates (3–14). Thus humans have an advantage over the other primates (14). By specialization of the brain function a various task can be accomplished at the same time (i.e., to speak and use tools). The decreased cerebral asymmetry as a part of abnormal neurodevelopment is proposed (3, 15, 16) to play a substantial role of the emergence of psychosis.

The eye dominance is the preference of one eye over the other, which allows the objects to be seen more clearly, more stable and even larger (17, 18). Thus perceptional processing priority is for the dominant eye (18).

According to neurodevelopmental hypothesis of schizophrenia an aberrant brain development during the fetal period leads to the disorder (19, 20). Lateralization of brain functions (including eye dominance) takes place in the early fetal stages of brain development, which suggests that cerebral asymmetry is a core feature of neurodevelopment.

Functional lateralization in schizophrenia is assessed mostly by hand or foot dominance, and occasionally by eye dominance. Data for eye dominance from the literature available to us seems very contradictory. Left eye dominance has been found (21–26) to be higher in schizophrenia patients vs. control subjects. Some authors (27, 28) have found higher left eye dominance only in male schizophrenia patients vs. control subjects but did not find such a relation in female schizophrenia patients. Besides, the reported findings of left eye dominance in schizophrenia patients are extremely variable. Some authors (28) have found as high as 73% left-eyedness (male schizophrenia subjects), while others (29, 30) did not find difference at all in eye dominance between schizophrenia patients and control subjects.

Minor physical anomalies (MPAs) can affect the head, the eyes, the ears, the mouth, the tongue as well as the torso and the limbs (31–33). The higher number of MPAs in schizophrenia patients than in control subjects ascertains the impaired early ontogenesis in this disorder (34–38). The first and/or early second trimester are critical for prenatal development and adverse events, respectively, anomalies in this period may serve as a key to understanding the important features of certain disruptions in ontogenesis.

In our study we apply four of the most popular and widely used instruments assessing handedness – Annett Hand Preference Questionnaire (AHPQ), Chapman and Chapman questionnaire, Edinburgh Handedness Inventory (EHI), Hand Preference Demonstration Test (HPDT). We used the 23 not overlapping tests from these scales and added 7 other hand tests, assessing the spontaneity. This made for a total number of 30 tests, selected to assess the proximal (shoulder, arm and forearm) and distal (wrist, palm and fingers) part of the upper limb in terms of quality of performance, precision and spontaneity. Despite the large number of tests, capturing several aspects of hand actions, and even though we used performance assessments, not preference questionnaires, we did not find statistically significant difference in left-handedness between schizophrenia patients and control subjects. In our opinion this result is not objective and is due to a specific cultural factor—the enforced right-hand writing during the communist regime till its collapse in 1990. Influences of cultural factors on hand dominance preference have been repeatedly documented (10, 22).

The preference of a dominant eye for vision is not affected by cultural influences and indicates more objectively brain lateralization. In this context, the aim of our study is to investigate the occurrence of left eye dominance and MPAs in schizophrenia patients and control subjects and to determine the relations between these two biological markers in schizophrenia.

## Subjects and methods

This study is a large project, which investigate six different markers (left-footedness, left-eyedness, left-handedness, digit ratio, minor physical anomalies, and cognitive (attention and memory) deficit) of neuronal dysontogenesis and the relations between them. I part of it will be presented in this article.

### Subjects

The study was conducted in the State Psychiatric Hospital in Radnevo and the Clinic of Psychiatry at the University Hospital in Sofia. The sample included 98 consecutively admitted in-patients with schizophrenia, 56 men and 42 women. The mean age was 34.45 years (SD = 15.67, range 23–79) for men and 42.20 years (SD = 11.38, range 21–63) for women.

The patients satisfied the Diagnostic and Statistical Manual of Mental Disorders-V (DSM-V) criteria for a diagnosis of schizophrenia based DSM-V semi-structured interview, case records and information obtained from relatives. Potential subjects, satisfying the above criteria for schizophrenia were excluded if they had a history of drug or alcohol abuse, identifiable neurological disorder (seizure disorder, head injury, multiple sclerosis, etc.), any signs of mental retardation or somatic disorder with neurological components in order to enhance the homogeneity of the schizophrenia patient group.

Impaired visual acuity, higher than ±2 diopters and more than ±1 diopter difference between the two eyes, any previous or present eye disorders were exclusion criteria, as these have been proven to confound the performance of the eye tasks.

The control group consisted of 82 subjects (30 men, 52 women) with a mean age 34.70 years (SD = 16.82, range 18–79) for men and 44.50 years (SD = 10.73, range 23–67) for women. Normality was defined as the absence of a psychiatric disorder. The control group satisfied the same exclusion criteria as those applied to the patients. In addition, to better separate control from schizophrenia group, exclusion criterion for controls was a first-degree relative with a history of a psychotic disorder, major affective disorder or suicide.

The schizophrenia patients group and the control group were of Bulgarian origin to avoid eventual racial or ethnic confound due to differences related to MPAs or lateralization. Any individual with other than Bulgarian parent or grandparent was also excluded.

The refusal rate of potential participants was insignificant (below 5%), excluding selectivity bias.

The study was approved by the Local Ethics Committee (Medical University of Sofia Scientific Ethics Committee) and all subjects provided written informed consent prior to participation.

### Instruments

Eye dominance (ocular sighting dominance) was measured by a battery of three tests—looking through monocle, hole-in-card test and Porta test.

Each test was administered as performance assessments, not as preference questionnaires. The tests were performed twice and if there was any inconsistency in preference, the subject was asked to perform the test again.

1. Looking through a monocle test—the participant is asked to take with both hands a monocle and look through it.
2. Hole-in-card test—the participant is asked to hold the card with both hands at arm’s length and look with both eyes through the hole at an object. While continuing to focus on the object and keeping the object centered in the hole with both eyes open, the participant has to slowly bring the card towards the face until it touches his nose. The card is positioned over one of the subject eyes.
3. Porta test (modified Miles test)—the participant is asked to extend one arm and to align a forefinger with a distant object with both eyes open, then to close his left eye and then the right eye consequently. The object is still aligned with the index finger when seeing with the dominant eye. Potential limitation of the test is the impact of the arm that the patient uses according to his hand dominance.

Each eye test is rated: 0 – Preference of right eye; 1 – No preference (both eyes equally); 2 – Preference of left eye. Each test score ranges from 0 to 2; the eye set total score ranges from 0 to 6.

The subjects were examined with a set of seven MPAs based on four items from the Waldrop Physical Anomaly Scale (39): Transversally furrowed tongue, Adherent earlobes, Single transverse palmar crease, Curved fifth finger. The last three variables were separately assessed for the left and for the right side and thus overall seven MPAs were assessed. We made modifications of some of the items: the categories Adherent earlobes and Lower edges of the ears extending backwards/upwards, which had two grades of a single item in the original scale, were defined as separate items because of the high prevalence of the first and the occasional finding of the second. In the original scale Radially curved fifth finger (clinodactyly) is measured in three degrees: 0 – Norm; 1 – Slightly radially curved; 2 – Strongly radially curved. We measured it in only two degrees: 0 – Absent or 1 – Present, due to the vague definition of “slightly” and “strongly” and the low Cohen’s *κ* < 0.60 of clinodactyly in the reliability study (40). Our acceptable level of reliability for concordance between categorical/ordinal scores was Cohen’s *κ* > 0.75.

Thus, the seven MPAs were scored qualitatively as 0 – Absent or 1 – Present. The score range for the MPA set was from 0 to 7.

All assessments were performed by the same examiner (K.A.).

### Statistical analysis

Data was analyzed with SPSS 25.0.

Descriptive statistics, various parametric and non-parametric tests were used.

The non-parametric Mann–Whitney test for means difference between two independent groups and the non-parametric Spearman’s rank correlation coefficient for correlation analysis were used, because our data is not continuous and lacks normal distribution.

The categories of the eye tests could be treated as ordinal data—graded, in ascending order of left-eyedness: 0 – Preference of right eye; 1 – No preference (both eyes equally); 2 – Preference of left eye. Ordinal data allows calculating the mean left-eyedness for every single eye test. A variable mean is usually more sensitive than its categories in detecting a difference between the groups. Besides that it enables comparing the important difference in the mean sum of left-eyedness of the three eye tests as a whole between the schizophrenia patients and the control subjects.

χ^2^- test (in 2 × 2 table with Yates’ correction for continuity) and odds ratio were used for comparing categorical data.

Multivariate analysis of variance (MANOVA) was used for comparing differences between several dependent variables overall.

Cluster analysis (K-Means Cluster Analysis) was used to determine if the three eye assessments could validly distinguish between the schizophrenia patients and the control subjects.

Statistical significance was defined as *p* < 0.05; two-tailed.

## Results

### Comparison of eye dominance between schizophrenia patients and control subjects

#### Comparison of each eye test

The frequency of left eye preference is significantly higher in schizophrenia patients than in controls in each of the three eye tests – Looking through a monocle (40.0% vs. 19.5%, *p* < 0.004), Hole test (42.1% vs. 19.5%, *p* < 0.003) and Porta test (42.4% vs. 20.7%, *p* < 0.006) (Table 1).

**Table 1:** Comparison of each eye dominance test between schizophrenic and control subjects.

	Schizophrenia (*N* = 98)*		Controls (*N* = 82)		Statistical significance**		Odds ratio	
Eye tests	*n*	%	*n*	%	*χ* ^2^	*p*	OR	95% CI
								
Looking through monocle					11.07	**0.004**	**3.00**	1.52–5.92
Right	55	57.9%	66	80.5%				
Both	2	2.1%	0	0.0%				
Left	38	40.0%	16	19.5%				
Hole test					11.59	**0.003**	**3.13**	1.58–6.18
Right	54	56.8%	66	80.5%				
Both	1	1.1%	0	0.0%				
Left	40	42.1%	16	19.5%				
Porta test					10.32	**0.006**	**2.95**	1.48–5.85
Right	48	56.5%	65	79.3%				
Both	1	1.2%	0	0.0%				
Left	36	42.4%	17	20.7%				

Bold values means statistical significance.

^*^Valid cases—the sum of the Right, Both, and Left categories does not always add up to 98, due to missing data for some cases.

^**^Pearson chi-square.

To calculate odds ratio (OR) the ordinal variables (scores 0, 1, and 2) of the eye tests were transformed into dichotomous variables (0 and 1): 0 – performing with the right eye; 1 – performing with no preferred eye or with the left eye. For the three eye tests, the 95% confidence interval of OR does not include the value of 1.00, indicating a statistically significant OR. Thus, the ORs show that performing the Hole test with the left or with both eyes is 3.13 times more frequent in schizophrenia patients than in control subjects; Looking through a monocle test—3.00 times more frequent and Porta test—2.95 times more frequent (Table 1).

#### Comparison of the sum of three eye tests

The total score of the three eye tests ranges from 0 to 6. The difference of the distribution of this sum between schizophrenia patients and control subjects is statistically significant (*p* < 0.005). The highest score of 6 shows the maximum leftward shift for eye dominance. It is noteworthy that over 3 times more schizophrenia patients than control subjects display this highest score—19 (22.4%) vs. 6 (7.3%). On the other hand, 1.7 times more control subjects than schizophrenia patients have the lowest sum 0, which indicates exclusive right-eyedness—55 (67.1%) vs. 33 (38.8%). Importantly, the small number of subjects with no eye preference (category “Both”) are entirely in the schizophrenia patients group (Table 2).

**Table 2:** Comparison of sum of three eye dominance tests between schizophrenic and control subjects.

	Schizophrenia (*N* = 85)*		Controls (*N* = 82)		Statistical significance*	
Eye tests	*n*	%	*n*	%	*χ* ^2^	*p*
Sum of 3 tests					16.53	**0.005**
6	19	**22.4%**	6	**7.3%**		
5	1	1.2%	0	0.0%		
4	12	14.1%	10	12.2%		
3	1	1.2%	0	0.0%		
2	19	22.4%	11	13.4%		
0	33	**38.8%**	55	**67.1%**		

Bold values means percent from all subjects and statistical significance.

^*^Pearson chi-square.

#### Comparison of the means

The mean of each eye test is approximately two times bigger in schizophrenia patients vs. control subjects and is statistically significantly: Looking through a hole—0.81 vs. 0.39, *p* < 0.001, over two times increase; Looking through a monocle—0.78 vs. 0.39, *p* < 0.002, two times increase; Porta test—0.86 vs. 0.41, *p* < 0.002, over two times increase (Table 3). Importantly, the mean sum of the three eye tests is more than two times bigger in schizophrenia patients than in control subjects and this difference shows marked statistical significance—2.45 vs. 1.20, *p* < 0.000, over two times increase.

**Table 3:** Comparison of mean left-eyedness between schizophrenic patients and control subjects.

	Schizophrenia (*n* = 95)		Controls (*n* = 82)		Statistical significance*	
Mean	Mean	SD	Mean	SD	*U*	*p*
Looking through monocle	0.78	0.97	0.39	0.80	3031.0	**0.002**
Hole test	0.81	0.98	0.39	0.80	2982.0	**0.001**
Porta test	0.86	0.99	0.41	0.82	2699.0	**0.002**
Mean sum of three tests	2.45	2.38	1.20	1.93	2418.0	**0.000**

Bold values means statistical significance.

* Mann–Whitney test.

### Comparison by using MANOVA and ANOVA

Multivariate Analysis of Variance (MANOVA) was used to study the effect of schizophrenia on the set of the three eye dominance tests. Using ANOVA for each eye test separately ignore the correlations or partial correlations between variables, thus a substantial information may be lost. MANOVA model takes into account only the “unique” contribution of every eye test of the set, adjusting for the effects of the other eye tests on every eye test. In our MANOVA model the set of three eye dominance tests were *multiple dependent variables* and the *independent classification variable* were the categories “schizophrenia patients and controls.” The most powerful and robust statistics for evaluating multivariate differences- the Pillai’s trace in this model is statistically significant (*F* = 4.74; *p* = 0.003; Table 4). This indicates a strong overall difference between schizophrenia patients and control subjects in the set of three eye dominance tests as a whole.

**Table 4:** MANOVA multivariate tests^b^.

Effect		Value	*F*	Hypothesis df	Error df	*p*
Schizophrenic vs. control subjects	Pillai’s trace	0.080	4,736^a^	3,000	163,000	0.003
	Wilks’ lambda	0.920	4,736^a^	3,000	163,000	0.003
	Hotelling’s trace	0.087	4,736^a^	3,000	163,000	0.003
	Roy’s largest root	0.087	4,736^a^	3,000	163,000	0.003

a Exact statistic.

b Design: Intercept + difference between Schizophrenic and Control Subjects.

The univariate ANOVA statistics with classification variable “schizophrenia patients vs. controls” and dependent variable—each single eye test shows statistically significant differences in favor of schizophrenia patients vs. control subjects for the three tests: Looking through a monocle—*F* = 10.10; *p* < 0.002; Looking through a hole—*F* = 11.48; *p* = 0.001; Porta test—*F* = 9.98; *p* < 0.002 (Table 5). The significance levels for the univariate ANOVA statistics are not adjusted for the fact that several comparisons are being made, therefore they have to be interpreted with caution. However, they indicate the individual contribution of every eye test to the overall statistical significance of the set of three eye dominance tests. The three eye tests separately contribute almost equally to the overall difference in left-eyedness between schizophrenia patients and control subjects, with Looking through a hole test showing slightly bigger contribution than the other two tests.

**Table 5:** Univariate analysis of variance—tests of between-subjects effects.

Dependent variable source	Type III sum of squares	df	Mean square	*F*	*p*
Looking through monocle*	8,168	1	8,168	10,104	0.002
Hole test**	9,410	1	9,410	11,479	0.001
Porta test***	8,235	1	8,235	9,975	0.002

* *R*^2^ = 0.055 (Adjusted R^2^ = 0.049).

** *R*^2^ = 0.062 (Adjusted *R*^2^ = 0.056).

*** *R*^2^ = 0.057 (Adjusted *R*^2^ = 0.051).

### Cluster analysis

The three eye tests (167 valid cases) were divided into two groups (clusters) in the end cluster centers by the K-Means Cluster Analysis (Table 6).

**Table 6:** End cluster centers.

	Looking through a monocle	Hole test	Porta test
Cluster 1	0.09	0.11	0.31
Cluster 2	1.78	1.80	1.45

One cluster center (cluster 1) has low values, while the other cluster center (cluster 2) has high values for left- eyedness of the three eye dominance tests: Hole test, Looking through a monocle and Porta test. The cross-tabulation of group *affiliation* “*Schizophrenia* vs. *Control subjects*” and “*Cluster 1* vs. *Cluster 2*” *allocation* (Table 7) distinguishes statistically significantly (*p* < 0.010) between schizophrenia patients and controls, allocating most of the schizophrenia subjects to cluster 2 and most of the control subjects to cluster 1. In the cluster with high value of left-eyedness (cluster 2) are allocated 49 subjects. The number of schizophrenia patients among them is more than twice the number of control subjects (67.3% vs. 32.7%). The opposite is presented in the cluster with low values of left-eyedness (cluster 1) with 118 subjects, where the control subjects are more than schizophrenia patients (55.9% vs. 44.1%).

**Table 7:** Cross-tabulation between group affiliation “schizophrenic patients vs. control subjects” and cluster allocation.

	Controls (*n* = 82)		Schizophrenia (*n* = 85)		Statistical significance*		
	*n*	%	*n*	%	*χ* ^2^	df	*p*
					6.605	1	**0.010**
Cluster 1	66	55.9	52	44.1			
Cluster 2	16	32.7	33	67.3			

Bold values means statistical significance.

^*^χ^2^- test in 2 × 2 table—Yates’ correction for continuity.

### Correlation between three eye tests

The nonparametric correlations between the three Eye Dominance Tests and between each Eye Dominance Test and the Sum of these Tests in the whole sample (schizophrenic and control subjects) are very strong (Table 8). All of them show high statistical significance (*p* < 0.01).

**Table 8:** Non-parametric Spearman’s rho correlations between three eye dominance tests and the sum of these tests in the whole sample (schizophrenic and control subjects).

		Looking through monocle	Hole test	Porta test	Sum of 3 tests
Looking through monocle	Correlation coefficient	1,000	0.722^**^	0.420^**^	0.825^**^
	Sig. (2-tailed)	–	**0.000**	**0.000**	**0.000**
	*N*	177	177	167	167
Hole test	Correlation coefficient	0.722^**^	1,000	0.403^**^	0.823^**^
	Sig. (2-tailed)	**0.000**	**–**	**0.000**	**0.000**
	*N*	177	177	167	167
Porta test	Correlation coefficient	0.420^**^	0.403^**^	1,000	0.763^**^
	Sig. (2-tailed)	**0.000**	**0.000**	**–**	**0.000**
	*N*	167	167	167	167
Sum of 3 tests	Correlation coefficient	0.825**	0.823**	0.763**	1,000
	Sig. (2-tailed)	**0.000**	**0.000**	**0.000**	–
	*N*	167	167	167	167

Bold values means statistical significance.

** Correlation is significant at the 0.01 level (2-tailed).

### Correlations between minor physical anomalies and left eye dominance

All seven MPAs show higher frequencies in schizophrenia vs. control subjects. The greatest differences are found for Single transverse palmar crease of the left hand (*p* < 0.002), Transversally furrowed tongue (*p* < 0.006), Single transverse palmar crease of the right hand (*p* < 0.054). Importantly, the sum of the seven MPAs is strongly significantly higher (*p* < 0.003) in schizophrenia vs. control subjects.

In order to address the low internal consistency of the MPAs of the Waldrop Physical Anomaly Scale (41) and to capture more subtly and diversely the multiple correlations between left-eyedness and MPAs, the seven MPAs were divided into three groups in descending order of their differentiating strength between schizophrenia patients and control subjects.

1. Sum 3 MPAs = Single transverse palmar crease of the left hand, Transversally furrowed tongue and Single transverse palmar crease of the right hand.
2. Sum 5 MPAs = Sum 3 MPAs + left/right Adherent earlobes.
3. Sum 7 MPAs = Sum 5 MPAs + left/right Curved fifth finger.

### Correlation matrix between the three eye tests and the seven MPAs

Spearman’s rank correlation matrix shows that positive correlations are highly predominant for left-eyedness and MPAs, with multiple statistically significant correlations:

- *Looking through a monocle with Transversally furrowed tongue (p < 0.023) and Sum 3 MPAs (p < 0.035).*
- *Hole test with Sum 3 MPAs (p < 0.045), Sum 5 MPAs (p < 0.023) and Sum 7 MPAs (p < 0.037).*
- *Sum of 3 eye tests with Sum 3 MPAs (p < 0.050) and Sum 5 MPAs (p < 0.026).*

The analysis of the correlation matrix ascertains a strong trend that the more left-eyed the schizophrenia patient is, the more s/he is stigmatized with MPAs and vice versa.

## Discussion

Lateralization of brain functions (including eye dominance) and MPAs are set in the early prenatal stages of neurodevelopment, albeit by mechanisms still not completely clarified. This suggests that brain asymmetry and MPAs are key characteristics of normal and abnormal neurodevelopment.

We found a significantly higher left eye dominance in schizophrenia patients vs. control subjects by using three eye dominance tests. In our opinion, the use of performance assessments, instead of preference questionnaires, and applying tests that are not influenced by hand preference, contributed to the relatively high levels of detected left-eyedness in schizophrenia patients (over 40%) and control subjects (about 20%) in our sample. Besides that, we prove this scientific fact by using several statistical methods. The five different statistics used (Pearson Chi-Square, Odds Ratio, Mann–Whitney, MANOVA, K-Means Cluster Analysis) showed that the three eye tests demonstrate, with almost equally strong statistical significance (close to *p* < 0.001), that schizophrenia patients with dominant left eye exceed more than twice those with dominant left eye among the control subjects.

The frequencies of left-eyedness in our sample are higher, but generally consistent with other authors (25, 28), who have also found higher left-eyedness in schizophrenia patients vs. control subjects. Giotakos has found that 30.4% of schizophrenia male patients have left eye dominance, which is less than our results of 40% (Looking through a monocle), 42.1% (Hole test) and 42.4% (Porta test) in a both-gender sample, including approximately equal number of males and females (22).

Eyes are part of the brain structure (originating from the prosencephalon) and probably the most direct indicator of hemispheric lateralization. We may tentatively speculate that this may be the cause of the high frequency of left eye dominance in our schizophrenia patients subsample (over 40%). During the embryonic period and particularly during the first trimester of pregnancy the neuronal disc was formed from the ectoderm and eye dominance asymmetry is the result of the implementation of this precisely guided program. This means that impaired cerebral lateralization can be considered by its nature as a neurodevelopmental disorder, which in turn underlies the development of schizophrenia. Higher left eye dominance in schizophrenia is an indicator of aberrant neurodevelopment.

Hand dominance is still the most widely used means of assessing laterality. However, eye dominance is a much subtler indicator of altered hemispheric lateralization than foot and hand dominance. First, left hand writing is strongly culturally influenced, e.g., was under cultural pressure during the communist regime (before 1990). Second, human societies are predominantly right sided and social conformity imposes the use of the right hand. Third, the material world of appliances, devices, instruments etc. is overall adapted to right-handed individuals.

Such cultural pressures are less likely to impact foot preference and do not affect eye preference at all. The assessment of eye dominance provides an adequate comparability among different countries and societies, by eliminating the cultural confound, when investigating laterality in various neuroontogenetic disorders such as ADHD, autism, schizotypy, psychoses and affective disorders.

The reasons for these findings could be very complicated, but knowledge of the neurological bases of laterality could be useful in this context. Hand and foot motor areas are somatotopically arranged in the primary motor area of the cerebral cortex. Neuronal connectivity of the visual pathway is more intricate, running a long distance through the brain hemisphere to reach the primary visual cortex in the occipital lobe. Additionally, the cortical area (frontal eye field) adjacent to the face related area in the precentral gyrus plays a significant role in the control of eye movements and visual attention. Voluntary eye movements require the additive and active signal from the frontomedian regions including the frontal eye, the dorsolateral prefrontal cortex, the supplementary eye field, and the anterior cingulate cortex (42).

Concerning the differences in the cortical projections of the limbs and those of the eyes it is unlikely to have the same hemispheric specialization for the different types of dominance. The limbs have afferent and efferent connections with the contralateral hemisphere of the brain. It is different for the eye, where the afference from one eye is projected to both hemispheres, and the efference to the muscles of one eye originates in both hemispheres, due to bilateral corticonuclear projections. Hence, brain lateralization is more evident for the hands and legs, but far less evident for the eyes.

MPAs are imprints of early dysontogenetic processes, and they are not changed by the course of the disease (36). Previous findings (40) confirmed the significantly higher stigmatization with MPAs of schizophrenia patients vs. control subjects. In our current data, statistically significant, strong, positive correlations were found between left-eyedness and stigmatization with MPAs. It is evident that the more left-eyed the subject is the more s/he is stigmatized with MPAs and vice versa. Such a correlation has not been described by other authors in the literature available to us.

The limitations of our study include the following: the limited sample size, because of which our results may not be sensitive enough and may not be able to detect a large effect, meaning that we would need a larger sample in order to have more significant findings; this study is clinic based, not community based, which means that it includes only in-patients, suggesting that the patients are more acute and accordingly there is selectivity bias.

We have assessed only a subset of MPAs from the Waldrop scale, which does not differentiate the minor malformation from phenogenetic variants. A larger number of MPAs as well as larger number of schizophrenia patients and control subjects will probably replicate the results in a more convincing manner. Further studies exploring the relations between left eye dominance and MPAs are still needed in order to research the various aspects of hemispheric asymmetry and dysfunction in schizophrenia.

## Conclusion

In our data we could correlate the impaired neurodevelopment, ascertained by stigmatization with MPAs, to the impaired cerebral lateralization measured by increased left eye dominance. Although we assessed just a subset of the multiple МPAs described in the literature, we regard the plenty of significant, positive correlations between left-eyedness and MPAs in the correlation matrix of three eye tests and seven MPAs an important additional proof of the neuronal dysontogenesis in schizophrenia. On the continuum of neuroontogenetic disorders, any single biological marker may indicate a probable neurodevelopmental disturbance. However, the higher co-occurrence of two biological markers for disontogenesis—left-eyedness and MPAs—in one subject, becomes a stronger reliable index of underlying neurodevelopmental disorder.

## Data availability statement

The raw data supporting the conclusions of this article will be made available by the authors, without undue reservation.

## Ethics statement

The studies involving human participants were reviewed and approved by Local Ethics Committee (Medical University of Sofia Scientific Ethics Committee). The patients/participants provided their written informed consent to participate in this study.

## Author contributions

KA: conceptualization, methodology, data curation, and writing—original draft preparation, validation, investigation and resources. The author confirms being the sole contributor of this work and has approved it for publication.

## Conflict of interest

The author declares that the research was conducted in the absence of any commercial or financial relationships that could be construed as a potential conflict of interest.

## Publisher’s note

All claims expressed in this article are solely those of the authors and do not necessarily represent those of their affiliated organizations, or those of the publisher, the editors and the reviewers. Any product that may be evaluated in this article, or claim that may be made by its manufacturer, is not guaranteed or endorsed by the publisher.
